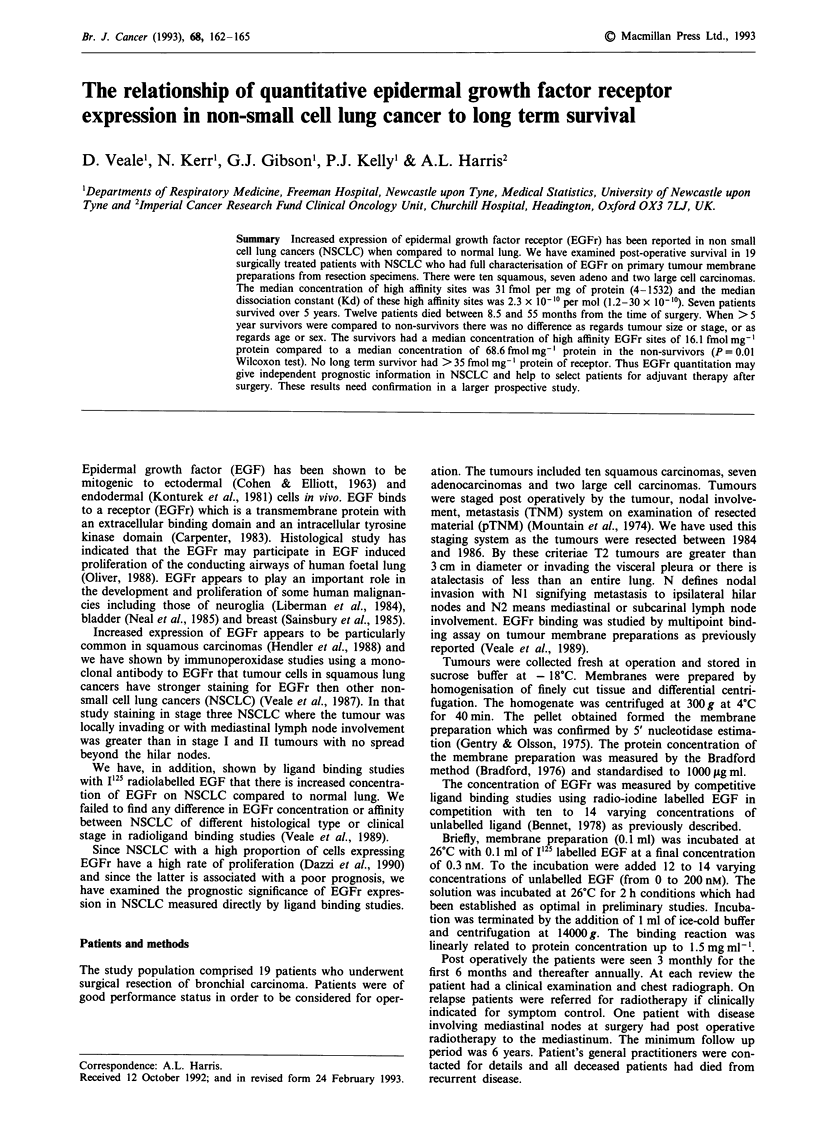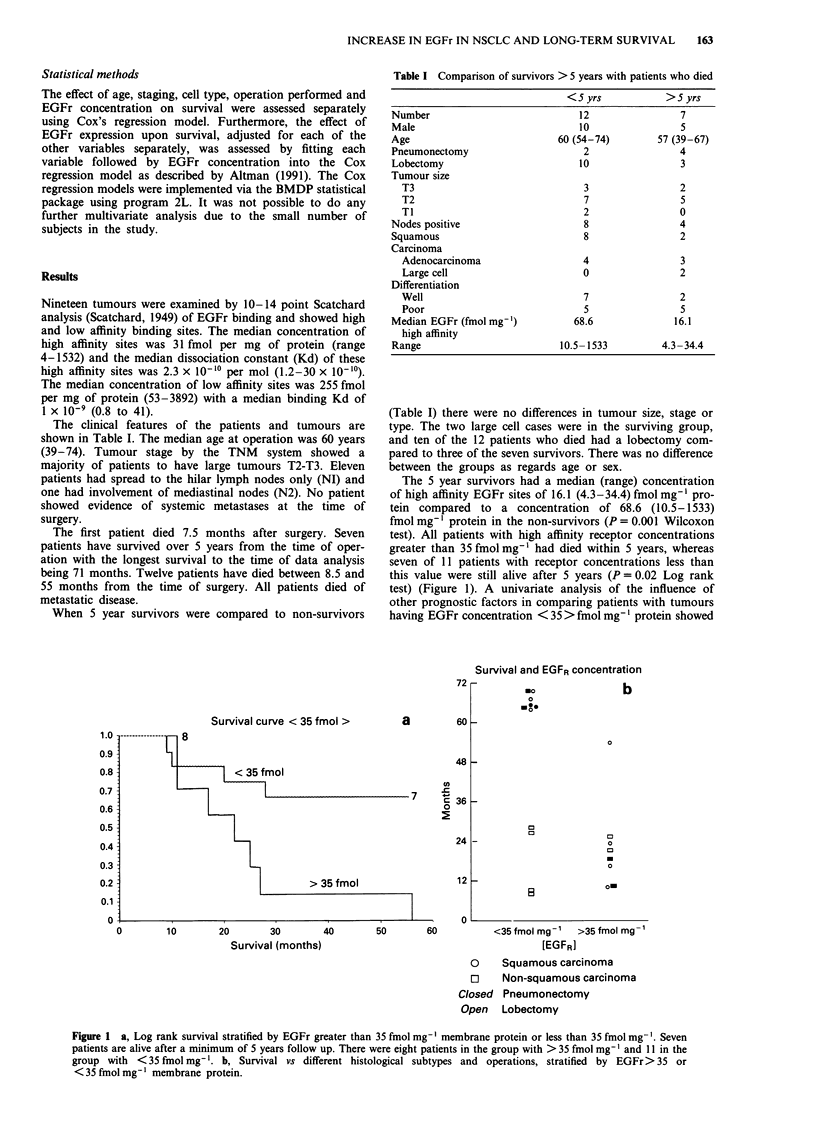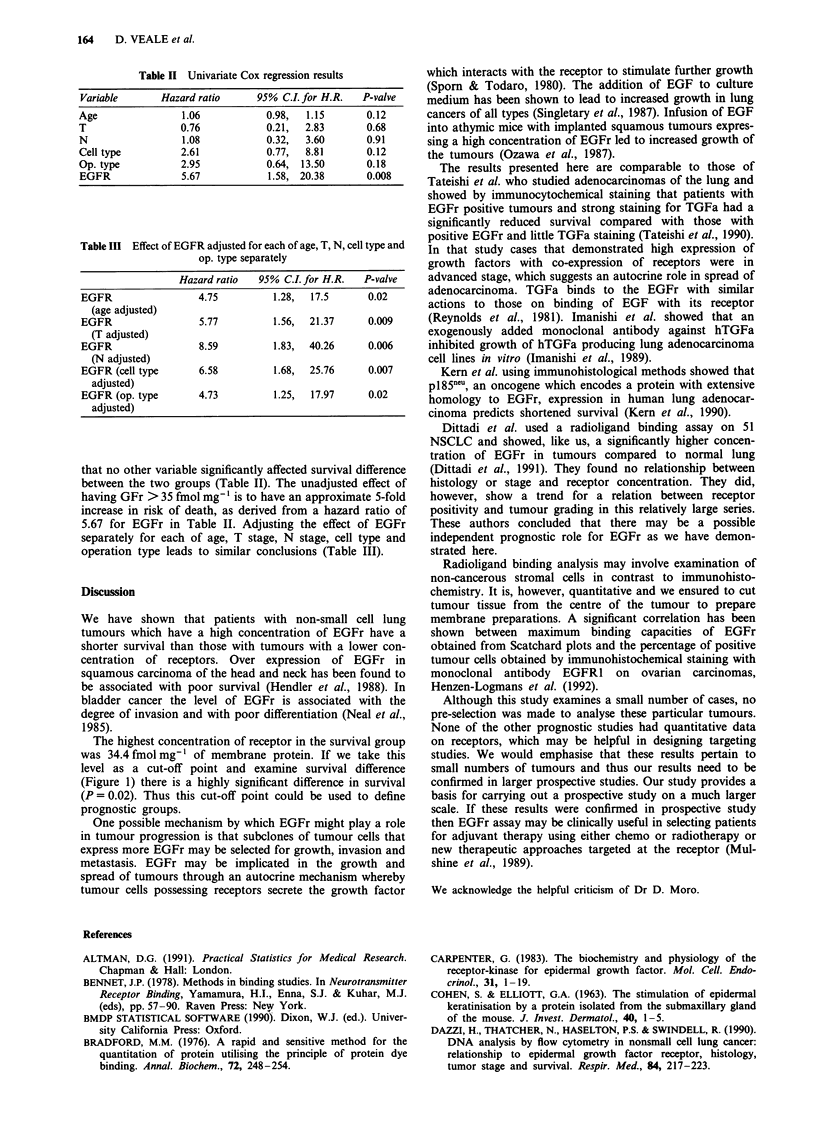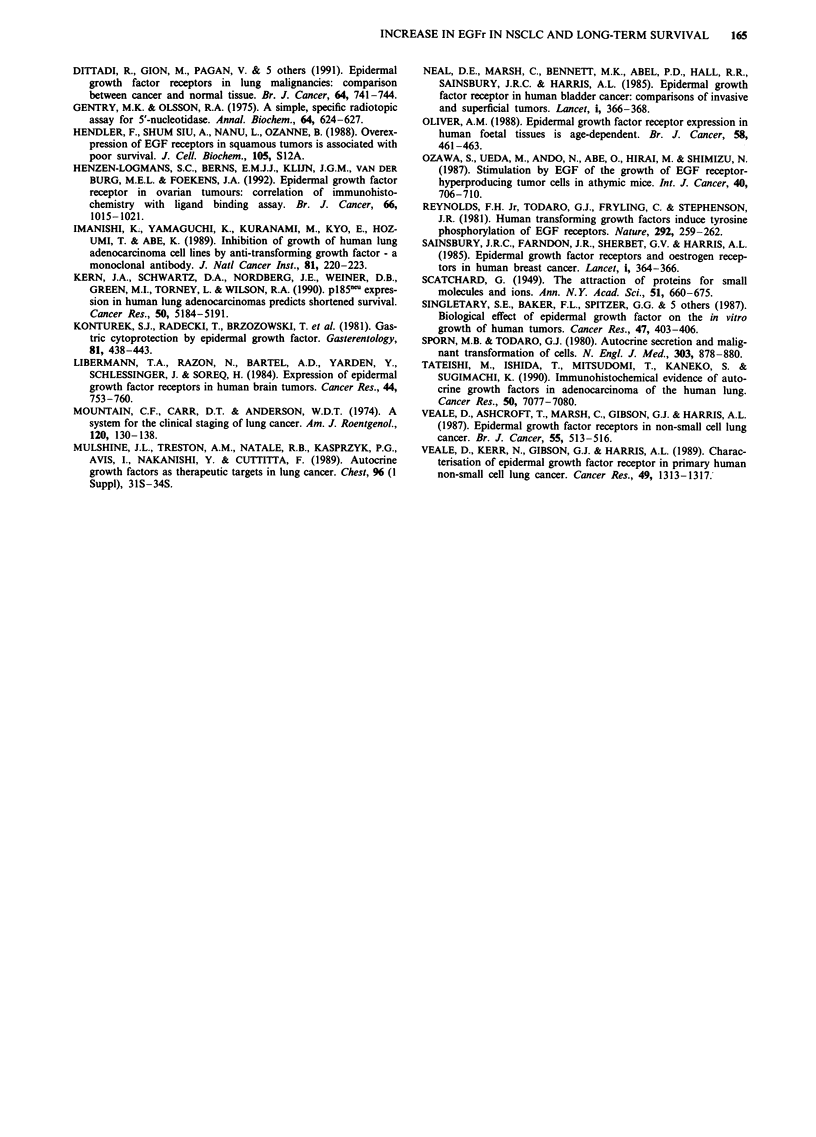# The relationship of quantitative epidermal growth factor receptor expression in non-small cell lung cancer to long term survival.

**DOI:** 10.1038/bjc.1993.306

**Published:** 1993-07

**Authors:** D. Veale, N. Kerr, G. J. Gibson, P. J. Kelly, A. L. Harris

**Affiliations:** Department of Respiratory Medicine, Freeman Hospital, Newcastle upon Tyne, UK.

## Abstract

Increased expression of epidermal growth factor receptor (EGFr) has been reported in non small cell lung cancers (NSCLC) when compared to normal lung. We have examined post-operative survival in 19 surgically treated patients with NSCLC who had full characterisation of EGFr on primary tumour membrane preparations from resection specimens. There were ten squamous, seven adeno and two large cell carcinomas. The median concentration of high affinity sites was 31 fmol per mg of protein (4-1532) and the median dissociation constant (Kd) of these high affinity sites was 2.3 x 10(-10) per mol (1.2-30 x 10(-10)). Seven patients survived over 5 years. Twelve patients died between 8.5 and 55 months from the time of surgery. When > 5 year survivors were compared to non-survivors there was no difference as regards tumour size or stage, or as regards age or sex. The survivors had a median concentration of high affinity EGFr sites of 16.1 fmol mg-1 protein compared to a median concentration of 68.6 fmol mg-1 protein in the non-survivors (P = 0.01 Wilcoxon test). No long term survivor had > 35 fmol mg-1 protein of receptor. Thus EGFr quantitation may give independent prognostic information in NSCLC and help to select patients for adjuvant therapy after surgery. These results need confirmation in a larger prospective study.


*Br. J. Cancer* (1993), **68**, 162–165 © Macmillan Press Ltd., 1993

### The relationship of quantitative epidermal growth factor receptor expression in non-small cell lung cancer to long term survival

D. Veale[1], N. Kerr[1], G.J. Gibson[1], P.J. Kelly[1] & A.L. Harris[2]

[1]*Departments of Respiratory Medicine, Freeman Hospital, Newcastle upon Tyne, Medical Statistics, University of Newcastle upon Tyne and* [2]*Imperial Cancer Research Fund Clinical Oncology Unit, Churchill Hospital, Headington, Oxford OX3 7LJ, UK.*

**Summary** Increased expression of epidermal growth factor receptor (EGFr) has been reported in non small cell lung cancers (NSCLC) when compared to normal lung. We have examined post-operative survival in 19 surgically treated patients with NSCLC who had full characterisation of EGFr on primary tumour membrane preparations from resection specimens. There were ten squamous, seven adeno and two large cell carcinomas. The median concentration of high affinity sites was 31 fmol per mg of protein (4–1532) and the median dissociation constant (Kd) of these high affinity sites was $2.3 \times 10^{-10}$ per mol ($1.2-30 \times 10^{-10}$). Seven patients survived over 5 years. Twelve patients died between 8.5 and 55 months from the time of surgery. When $>5$ year survivors were compared to non-survivors there was no difference as regards tumour size or stage, or as regards age or sex. The survivors had a median concentration of high affinity EGFr sites of 16.1 fmol $mg^{-1}$ protein compared to a median concentration of 68.6 fmol $mg^{-1}$ protein in the non-survivors ($P = 0.01$ Wilcoxon test). No long term survivor had $>35$ fmol $mg^{-1}$ protein of receptor. Thus EGFr quantitation may give independent prognostic information in NSCLC and help to select patients for adjuvant therapy after surgery. These results need confirmation in a larger prospective study.

Epidermal growth factor (EGF) has been shown to be mitogenic to ectodermal (Cohen & Elliott, 1963) and endodermal (Konturek *et al.*, 1981) cells *in vivo*. EGF binds to a receptor (EGFr) which is a transmembrane protein with an extracellular binding domain and an intracellular tyrosine kinase domain (Carpenter, 1983). Histological study has indicated that the EGFr may participate in EGF induced proliferation of the conducting airways of human foetal lung (Oliver, 1988). EGFr appears to play an important role in the development and proliferation of some human malignancies including those of neuroglia (Liberman *et al.*, 1984), bladder (Neal *et al.*, 1985) and breast (Sainsbury *et al.*, 1985).

Increased expression of EGFr appears to be particularly common in squamous carcinomas (Hendler *et al.*, 1988) and we have shown by immunoperoxidase studies using a monoclonal antibody to EGFr that tumour cells in squamous lung cancers have stronger staining for EGFr then other non-small cell lung cancers (NSCLC) (Veale *et al.*, 1987). In that study staining in stage three NSCLC where the tumour was locally invading or with mediastinal lymph node involvement was greater than in stage I and II tumours with no spread beyond the hilar nodes.

We have, in addition, shown by ligand binding studies with $I^{125}$ radiolabelled EGF that there is increased concentration of EGFr on NSCLC compared to normal lung. We failed to find any difference in EGFr concentration or affinity between NSCLC of different histological type or clinical stage in radioligand binding studies (Veale *et al.*, 1989).

Since NSCLC with a high proportion of cells expressing EGFr have a high rate of proliferation (Dazzi *et al.*, 1990) and since the latter is associated with a poor prognosis, we have examined the prognostic significance of EGFr expression in NSCLC measured directly by ligand binding studies.

### Patients and methods

The study population comprised 19 patients who underwent surgical resection of bronchial carcinoma. Patients were of good performance status in order to be considered for operation. The tumours included ten squamous carcinomas, seven adenocarcinomas and two large cell carcinomas. Tumours were staged post operatively by the tumour, nodal involvement, metastasis (TNM) system on examination of resected material (pTNM) (Mountain *et al.*, 1974). We have used this staging system as the tumours were resected between 1984 and 1986. By these criteriae T2 tumours are greater than 3 cm in diameter or invading the visceral pleura or there is atalectasis of less than an entire lung. N defines nodal invasion with N1 signifying metastasis to ipsilateral hilar nodes and N2 means mediastinal or subcarinal lymph node involvement. EGFr binding was studied by multipoint binding assay on tumour membrane preparations as previously reported (Veale *et al.*, 1989).

Tumours were collected fresh at operation and stored in sucrose buffer at − 18°C. Membranes were prepared by homogenisation of finely cut tissue and differential centrifugation. The homogenate was centrifuged at 300 *g* at 4°C for 40 min. The pellet obtained formed the membrane preparation which was confirmed by 5′ nucleotidase estimation (Gentry & Olsson, 1975). The protein concentration of the membrane preparation was measured by the Bradford method (Bradford, 1976) and standardised to 1000 μg ml.

The concentration of EGFr was measured by competitive ligand binding studies using radio-iodine labelled EGF in competition with ten to 14 varying concentrations of unlabelled ligand (Bennet, 1978) as previously described.

Briefly, membrane preparation (0.1 ml) was incubated at 26°C with 0.1 ml of $I^{125}$ labelled EGF at a final concentration of 0.3 nM. To the incubation were added 12 to 14 varying concentrations of unlabelled EGF (from 0 to 200 nM). The solution was incubated at 26°C for 2 h conditions which had been established as optimal in preliminary studies. Incubation was terminated by the addition of 1 ml of ice-cold buffer and centrifugation at 14000 *g*. The binding reaction was linearly related to protein concentration up to 1.5 mg $ml^{-1}$.

Post operatively the patients were seen 3 monthly for the first 6 months and thereafter annually. At each review the patient had a clinical examination and chest radiograph. On relapse patients were referred for radiotherapy if clinically indicated for symptom control. One patient with disease involving mediastinal nodes at surgery had post operative radiotherapy to the mediastinum. The minimum follow up period was 6 years. Patient's general practitioners were contacted for details and all deceased patients had died from recurrent disease.

Correspondence: A.L. Harris.
Received 12 October 1992; and in revised form 24 February 1993.

*Statistical methods*

The effect of age, staging, cell type, operation performed and EGFr concentration on survival were assessed separately using Cox's regression model. Furthermore, the effect of EGFr expression upon survival, adjusted for each of the other variables separately, was assessed by fitting each variable followed by EGFr concentration into the Cox regression model as described by Altman (1991). The Cox regression models were implemented via the BMDP statistical package using program 2L. It was not possible to do any further multivariate analysis due to the small number of subjects in the study.

### Results

Nineteen tumours were examined by 10–14 point Scatchard analysis (Scatchard, 1949) of EGFr binding and showed high and low affinity binding sites. The median concentration of high affinity sites was 31 fmol per mg of protein (range 4–1532) and the median dissociation constant (Kd) of these high affinity sites was $2.3 \times 10^{-10}$ per mol ($1.2-30 \times 10^{-10}$). The median concentration of low affinity sites was 255 fmol per mg of protein (53–3892) with a median binding Kd of $1 \times 10^{-9}$ (0.8 to 41).

The clinical features of the patients and tumours are shown in Table I. The median age at operation was 60 years (39–74). Tumour stage by the TNM system showed a majority of patients to have large tumours T2-T3. Eleven patients had spread to the hilar lymph nodes only (NI) and one had involvement of mediastinal nodes (N2). No patient showed evidence of systemic metastases at the time of surgery.

The first patient died 7.5 months after surgery. Seven patients have survived over 5 years from the time of operation with the longest survival to the time of data analysis being 71 months. Twelve patients have died between 8.5 and 55 months from the time of surgery. All patients died of metastatic disease.

When 5 year survivors were compared to non-survivors

**Table I** Comparison of survivors >5 years with patients who died

| | *<5 yrs* | *>5 yrs* |
|---|---|---|
| Number | 12 | 7 |
| Male | 10 | 5 |
| Age | 60 (54–74) | 57 (39–67) |
| Pneumonectomy | 2 | 4 |
| Lobectomy | 10 | 3 |
| Tumour size | | |
| T3 | 3 | 2 |
| T2 | 7 | 5 |
| T1 | 2 | 0 |
| Nodes positive | 8 | 4 |
| Squamous | 8 | 2 |
| Carcinoma | | |
| Adenocarcinoma | 4 | 3 |
| Large cell | 0 | 2 |
| Differentiation | | |
| Well | 7 | 2 |
| Poor | 5 | 5 |
| Median EGFr (fmol mg$^{-1}$) high affinity | 68.6 | 16.1 |
| Range | 10.5–1533 | 4.3–34.4 |

(Table I) there were no differences in tumour size, stage or type. The two large cell cases were in the surviving group, and ten of the 12 patients who died had a lobectomy compared to three of the seven survivors. There was no difference between the groups as regards age or sex.

The 5 year survivors had a median (range) concentration of high affinity EGFr sites of 16.1 (4.3–34.4) fmol mg$^{-1}$ protein compared to a concentration of 68.6 (10.5–1533) fmol mg$^{-1}$ protein in the non-survivors ($P = 0.001$ Wilcoxon test). All patients with high affinity receptor concentrations greater than 35 fmol mg$^{-1}$ had died within 5 years, whereas seven of 11 patients with receptor concentrations less than this value were still alive after 5 years ($P = 0.02$ Log rank test) (Figure 1). A univariate analysis of the influence of other prognostic factors in comparing patients with tumours having EGFr concentration <35> fmol mg$^{-1}$ protein showed

**Figure 1** **a**, Log rank survival stratified by EGFr greater than 35 fmol mg$^{-1}$ membrane protein or less than 35 fmol mg$^{-1}$. Seven patients are alive after a minimum of 5 years follow up. There were eight patients in the group with >35 fmol mg$^{-1}$ and 11 in the group with <35 fmol mg$^{-1}$. **b**, Survival *vs* different histological subtypes and operations, stratified by EGFr>35 or <35 fmol mg$^{-1}$ membrane protein.

Table II Univariate Cox regression results

| Variable | Hazard ratio | 95% C.I. for H.R. | P-valve |
|---|---|---|---|
| Age | 1.06 | 0.98, 1.15 | 0.12 |
| T | 0.76 | 0.21, 2.83 | 0.68 |
| N | 1.08 | 0.32, 3.60 | 0.91 |
| Cell type | 2.61 | 0.77, 8.81 | 0.12 |
| Op. type | 2.95 | 0.64, 13.50 | 0.18 |
| EGFR | 5.67 | 1.58, 20.38 | 0.008 |

Table III Effect of EGFR adjusted for each of age, T, N, cell type and op. type separately

| | Hazard ratio | 95% C.I. for H.R. | P-valve |
|---|---|---|---|
| EGFR (age adjusted) | 4.75 | 1.28, 17.5 | 0.02 |
| EGFR (T adjusted) | 5.77 | 1.56, 21.37 | 0.009 |
| EGFR (N adjusted) | 8.59 | 1.83, 40.26 | 0.006 |
| EGFR (cell type adjusted) | 6.58 | 1.68, 25.76 | 0.007 |
| EGFR (op. type adjusted) | 4.73 | 1.25, 17.97 | 0.02 |

that no other variable significantly affected survival difference between the two groups (Table II). The unadjusted effect of having GFr $>35$ fmol mg$^{-1}$ is to have an approximate 5-fold increase in risk of death, as derived from a hazard ratio of 5.67 for EGFr in Table II. Adjusting the effect of EGFr separately for each of age, T stage, N stage, cell type and operation type leads to similar conclusions (Table III).

### Discussion

We have shown that patients with non-small cell lung tumours which have a high concentration of EGFr have a shorter survival than those with tumours with a lower concentration of receptors. Over expression of EGFr in squamous carcinoma of the head and neck has been found to be associated with poor survival (Hendler et al., 1988). In bladder cancer the level of EGFr is associated with the degree of invasion and with poor differentiation (Neal et al., 1985).

The highest concentration of receptor in the survival group was 34.4 fmol mg$^{-1}$ of membrane protein. If we take this level as a cut-off point and examine survival difference (Figure 1) there is a highly significant difference in survival ($P = 0.02$). Thus this cut-off point could be used to define prognostic groups.

One possible mechanism by which EGFr might play a role in tumour progression is that subclones of tumour cells that express more EGFr may be selected for growth, invasion and metastasis. EGFr may be implicated in the growth and spread of tumours through an autocrine mechanism whereby tumour cells possessing receptors secrete the growth factor which interacts with the receptor to stimulate further growth (Sporn & Todaro, 1980). The addition of EGF to culture medium has been shown to lead to increased growth in lung cancers of all types (Singletary et al., 1987). Infusion of EGF into athymic mice with implanted squamous tumours expressing a high concentration of EGFr led to increased growth of the tumours (Ozawa et al., 1987).

The results presented here are comparable to those of Tateishi et al. who studied adenocarcinomas of the lung and showed by immunocytochemical staining that patients with EGFr positive tumours and strong staining for TGFa had a significantly reduced survival compared with those with positive EGFr and little TGFa staining (Tateishi et al., 1990). In that study cases that demonstrated high expression of growth factors with co-expression of receptors were in advanced stage, which suggests an autocrine role in spread of adenocarcinoma. TGFa binds to the EGFr with similar actions to those on binding of EGF with its receptor (Reynolds et al., 1981). Imanishi et al. showed that an exogenously added monoclonal antibody against hTGFa inhibited growth of hTGFa producing lung adenocarcinoma cell lines *in vitro* (Imanishi et al., 1989).

Kern et al. using immunohistological methods showed that $p185^{neu}$, an oncogene which encodes a protein with extensive homology to EGFr, expression in human lung adenocarcinoma predicts shortened survival (Kern et al., 1990).

Dittadi et al. used a radioligand binding assay on 51 NSCLC and showed, like us, a significantly higher concentration of EGFr in tumours compared to normal lung (Dittadi et al., 1991). They found no relationship between histology or stage and receptor concentration. They did, however, show a trend for a relation between receptor positivity and tumour grading in this relatively large series. These authors concluded that there may be a possible independent prognostic role for EGFr as we have demonstrated here.

Radioligand binding analysis may involve examination of non-cancerous stromal cells in contrast to immunohistochemistry. It is, however, quantitative and we ensured to cut tumour tissue from the centre of the tumour to prepare membrane preparations. A significant correlation has been shown between maximum binding capacities of EGFr obtained from Scatchard plots and the percentage of positive tumour cells obtained by immunohistochemical staining with monoclonal antibody EGFR1 on ovarian carcinomas, Henzen-Logmans et al. (1992).

Although this study examines a small number of cases, no pre-selection was made to analyse these particular tumours. None of the other prognostic studies had quantitative data on receptors, which may be helpful in designing targeting studies. We would emphasise that these results pertain to small numbers of tumours and thus our results need to be confirmed in larger prospective studies. Our study provides a basis for carrying out a prospective study on a much larger scale. If these results were confirmed in prospective study then EGFr assay may be clinically useful in selecting patients for adjuvant therapy using either chemo or radiotherapy or new therapeutic approaches targeted at the receptor (Mulshine et al., 1989).

We acknowledge the helpful criticism of Dr D. Moro.

### References

ALTMAN, D.G. (1991). *Practical Statistics for Medical Research*. Chapman & Hall: London.

BENNET, J.P. (1978). Methods in binding studies. In *Neurotransmitter Receptor Binding*, Yamamura, H.I., Enna, S.J. & Kuhar, M.J. (eds), pp. 57–90. Raven Press: New York.

BMDP STATISTICAL SOFTWARE (1990). Dixon, W.J. (ed.). University California Press: Oxford.

BRADFORD, M.M. (1976). A rapid and sensitive method for the quantitation of protein utilising the principle of protein dye binding. *Annal. Biochem.*, **72**, 248–254.

CARPENTER, G. (1983). The biochemistry and physiology of the receptor-kinase for epidermal growth factor. *Mol. Cell. Endocrinol.*, **31**, 1–19.

COHEN, S. & ELLIOTT, G.A. (1963). The stimulation of epidermal keratinisation by a protein isolated from the submaxillary gland of the mouse. *J. Invest. Dermatol.*, **40**, 1–5.

DAZZI, H., THATCHER, N., HASELTON, P.S. & SWINDELL, R. (1990). DNA analysis by flow cytometry in nonsmall cell lung cancer: relationship to epidermal growth factor receptor, histology, tumor stage and survival. *Respir. Med.*, **84**, 217–223.

DITTADI, R., GION, M., PAGAN, V. & 5 others (1991). Epidermal growth factor receptors in lung malignancies: comparison between cancer and normal tissue. *Br. J. Cancer*, **64**, 741–744.

GENTRY, M.K. & OLSSON, R.A. (1975). A simple, specific radiotopic assay for 5'-nucleotidase. *Annal. Biochem.*, **64**, 624–627.

HENDLER, F., SHUM SIU, A., NANU, L., OZANNE, B. (1988). Overexpression of EGF receptors in squamous tumors is associated with poor survival. *J. Cell. Biochem.*, **105**, S12A.

HENZEN-LOGMANS, S.C., BERNS, E.M.J.J., KLIJN, J.G.M., VAN DER BURG, M.E.L. & FOEKENS, J.A. (1992). Epidermal growth factor receptor in ovarian tumours: correlation of immunohistochemistry with ligand binding assay. *Br. J. Cancer*, **66**, 1015–1021.

IMANISHI, K., YAMAGUCHI, K., KURANAMI, M., KYO, E., HOZUMI, T. & ABE, K. (1989). Inhibition of growth of human lung adenocarcinoma cell lines by anti-transforming growth factor - a monoclonal antibody. *J. Natl Cancer Inst.*, **81**, 220–223.

KERN, J.A., SCHWARTZ, D.A., NORDBERG, J.E., WEINER, D.B., GREEN, M.I., TORNEY, L. & WILSON, R.A. (1990). $p185^{neu}$ expression in human lung adenocarcinomas predicts shortened survival. *Cancer Res.*, **50**, 5184–5191.

KONTUREK, S.J., RADECKI, T., BRZOZOWSKI, T. *et al.* (1981). Gastric cytoprotection by epidermal growth factor. *Gasterentology*, **81**, 438–443.

LIBERMANN, T.A., RAZON, N., BARTEL, A.D., YARDEN, Y., SCHLESSINGER, J. & SOREQ, H. (1984). Expression of epidermal growth factor receptors in human brain tumors. *Cancer Res.*, **44**, 753–760.

MOUNTAIN, C.F., CARR, D.T. & ANDERSON, W.D.T. (1974). A system for the clinical staging of lung cancer. *Am. J. Roentgenol.*, **120**, 130–138.

MULSHINE, J.L., TRESTON, A.M., NATALE, R.B., KASPRZYK, P.G., AVIS, I., NAKANISHI, Y. & CUTTITTA, F. (1989). Autocrine growth factors as therapeutic targets in lung cancer. *Chest*, **96** (1 Suppl), 31S–34S.

NEAL, D.E., MARSH, C., BENNETT, M.K., ABEL, P.D., HALL, R.R., SAINSBURY, J.R.C. & HARRIS, A.L. (1985). Epidermal growth factor receptor in human bladder cancer: comparisons of invasive and superficial tumors. *Lancet*, **i**, 366–368.

OLIVER, A.M. (1988). Epidermal growth factor receptor expression in human foetal tissues is age-dependent. *Br. J. Cancer*, **58**, 461–463.

OZAWA, S., UEDA, M., ANDO, N., ABE, O., HIRAI, M. & SHIMIZU, N. (1987). Stimulation by EGF of the growth of EGF receptor-hyperproducing tumor cells in athymic mice. *Int. J. Cancer*, **40**, 706–710.

REYNOLDS, F.H. Jr, TODARO, G.J., FRYLING, C. & STEPHENSON, J.R. (1981). Human transforming growth factors induce tyrosine phosphorylation of EGF receptors. *Nature*, **292**, 259–262.

SAINSBURY, J.R.C., FARNDON, J.R., SHERBET, G.V. & HARRIS, A.L. (1985). Epidermal growth factor receptors and oestrogen receptors in human breast cancer. *Lancet*, **i**, 364–366.

SCATCHARD, G. (1949). The attraction of proteins for small molecules and ions. *Ann. N.Y. Acad. Sci.*, **51**, 660–675.

SINGLETARY, S.E., BAKER, F.L., SPITZER, G.G. & 5 others (1987). Biological effect of epidermal growth factor on the *in vitro* growth of human tumors. *Cancer Res.*, **47**, 403–406.

SPORN, M.B. & TODARO, G.J. (1980). Autocrine secretion and malignant transformation of cells. *N. Engl. J. Med.*, **303**, 878–880.

TATEISHI, M., ISHIDA, T., MITSUDOMI, T., KANEKO, S. & SUGIMACHI, K. (1990). Immunohistochemical evidence of autocrine growth factors in adenocarcinoma of the human lung. *Cancer Res.*, **50**, 7077–7080.

VEALE, D., ASHCROFT, T., MARSH, C., GIBSON, G.J. & HARRIS, A.L. (1987). Epidermal growth factor receptors in non-small cell lung cancer. *Br. J. Cancer*, **55**, 513–516.

VEALE, D., KERR, N., GIBSON, G.J. & HARRIS, A.L. (1989). Characterisation of epidermal growth factor receptor in primary human non-small cell lung cancer. *Cancer Res.*, **49**, 1313–1317.